# Reflections, impact and recommendations of a co‐produced qualitative study with young people who have experience of mental health difficulties

**DOI:** 10.1111/hex.13088

**Published:** 2020-06-09

**Authors:** Lindsay H. Dewa, Anna Lawrence‐Jones, Caroline Crandell, Jack Jaques, Katy Pickles, Mary Lavelle, Sofia Pappa, Paul Aylin

**Affiliations:** ^1^ NIHR Patient Safety Translational Research Centre Imperial College London London UK; ^2^ School of Public Health Imperial College London London UK; ^3^ The McPin Foundation London UK; ^4^ City, University of London London UK; ^5^ West London NHS Trust London UK

**Keywords:** co‐production, health research, mental health, patient and public involvement, technology, young people

## Abstract

**Background:**

There is limited evidence of genuine equal partnership where power is shared with young people with mental health difficulties throughout all research stages, particularly in data collection and analysis.

**Objective:**

To describe how our qualitative study, exploring young peoples’ perceptions on the feasibility of using technology to detect mental health deterioration, was co‐produced using principles of co‐production, whilst reflecting on impact, challenges and recommendations.

**Methods:**

Young people with experience of mental health difficulties were appointed and then worked with researchers throughout all research stages. The study was evaluated against the five principles of co‐production. Reflections from researchers and young people were collected throughout.

**Results:**

Seven young people formed an initial Young People's Advisory Group (YPAG); three became co‐researchers. Reflection was key throughout the process. Sharing power became easier and more evident as trust, confidence and mutual respect grew over time, particularly after a safe space was established. The safe space was crucial for open discussions, and our WhatsApp group enabled continual communication, support and shared decision‐making. The resulting co‐produced topic guide, coding framework, thematic map, papers and presentations demonstrated significant impact.

**Conclusions:**

To our knowledge, this is the first qualitative mental health study to be co‐produced using the principles of co‐production. Our rigorous assessment can be utilized as an informative document to help others to produce meaningful co‐produced future research. Although co‐production takes time, it makes significant impact to the research, researchers and co‐researchers. Flexible funding for spontaneous suggestions from co‐researchers and more time for interview training is recommended.

## 1 BACKGROUND

Patient and public involvement (PPI) is defined as research *with* and *by* patients, rather than *to, for* or *about* them.^1^ In recent years, there has been a significant shift in PPI in research, moving away from tokenism, towards more meaningful involvement and co‐production.^2^ This has been facilitated by changes in research infrastructure and the growing patient voice. Increasingly, funding bodies have made it compulsory for prospective applicants to declare how patients, carers or the public have and will be involved in the proposed research. Likewise, some journals also require researchers to add a PPI statement^3^, ^4^ to their manuscript. This has led to research organizations creating dedicated PPI resources within their institutions.

Patients, carers and members of the public can be co‐researchers and equal partners throughout the whole research process: ideas generation, design, applying for funding, ethics, management, data collection, analysis, evaluation and dissemination.^5^ Some peer‐reviewed papers have discussed the benefits, costs and learning of co‐production,^6^, ^7^ and some larger studies have found positive impact on findings (eg shared decision‐making in mental health services).^8^, ^9^ However, most studies have not been evaluated against NIHR INVOLVE’s principles of co‐production. There are five main principles: sharing power, including all perspectives and skills, respecting and valuing the knowledge of all those working together, reciprocity, and building and maintaining relationships.^10^ Critically, whilst these principles help guide our work, they do not tell us how to co‐produce research, the main challenges that might arise, or how to overcome these challenges.^11^ INVOLVE have recently produced two reports that showcase selected studies against the principles, and on how to co‐produce research.^12^, ^13^ However, none are focused on young people with experience of mental health difficulties. Indeed, there is limited evidence of genuine equal partnership where power is shared throughout project stages in mental health research.

The systematic evaluation of PPI started almost ten years ago,^14^ but it is only recently that capturing the impact of involvement has been formalized. For example, The BMJ and the National Institute for Health Research (NIHR) now suggest researchers use the GRIPP2 checklist^15^ to report on PPI. Numerous papers have shown value in involving people from differing population groups, through various research stages and in different research designs.^16^, ^17^, ^18^, ^19^ However, there is an ongoing debate in the field as to if and how the impact of PPI should be captured or measured. Many of the existing tools are fairly prescriptive and lead to more quantitative measurements of who and how people were involved, rather than qualitative reflection (from all parties), on what has changed due to the involvement. PPI is ultimately about an interaction between researchers and patients or members of the public, which is dynamic, context specific and dependent upon many uncontrollable factors.^20^

There is also a significant research gap in working with young people throughout all stages of a mental health research project and evaluating this involvement. We recently conducted a qualitative study that explored the acceptability and feasibility of technologies to detect mental health deterioration in young people with current mental health difficulties, where young people were research partners.^21^ This research topic was informed by the McPin Foundation Priority Setting Partnership (PSP) on children and young people's mental health, in partnership with James Lind Alliance (JLA).^22^ The PSP was rigorous process where children, young people, teachers, parents, mental health professionals and researchers came up with and prioritized research questions. Our study was therefore co‐produced from start to end, ensuring it was appropriately designed, delivered and impactful.^23^ The aim of this article is to: (a) describe how our study was co‐produced following the principles of co‐production; and (b) report on reflections that capture the impact on the research, researchers and co‐researchers. The main challenges that arose and advice on how to overcome them in similarly co‐produced studies are also described. This paper was reported using the GRIPP2 checklist (Appendix 1).^15^

## 2 METHOD

### 2.1 Appointment of young co‐researchers

The opportunity to work on our project^21^ was advertised through a variety of methods, including: Twitter, ‘People in Research’ website and The McPin Foundation newsletter. The McPin Foundation PPI lead also disseminated the advertisement to approximately 100 young people listed on their PPI database via email. After an internal application review by the lead researcher (LD) and our PPI lead (ALJ), seven young people (aged 18‐25) were appointed to the Young People's Advisory Group (YPAG) based on relevance to the topic (eg lived experience of mental health difficulties, use of technology and interest in research). The YPAG was split across gender (n = 2 male) and ethnicities (n = 5 White‐British; n = 1 British‐Asian; and n = 1 Black‐British), and had lived experience of mental health difficulties including depression, anxiety disorder, bipolar disorder, anorexia nervosa, psychosis, substance misuse and personality disorder. Experience of technologies and research varied but all had used social media, mobile apps and websites. All reflections were captured throughout using Evernote, a digital app that helps manage notes, ideas and photographs across different users and devices.^24^

### 2.2 Design and ethics

**FIGURE 1:**
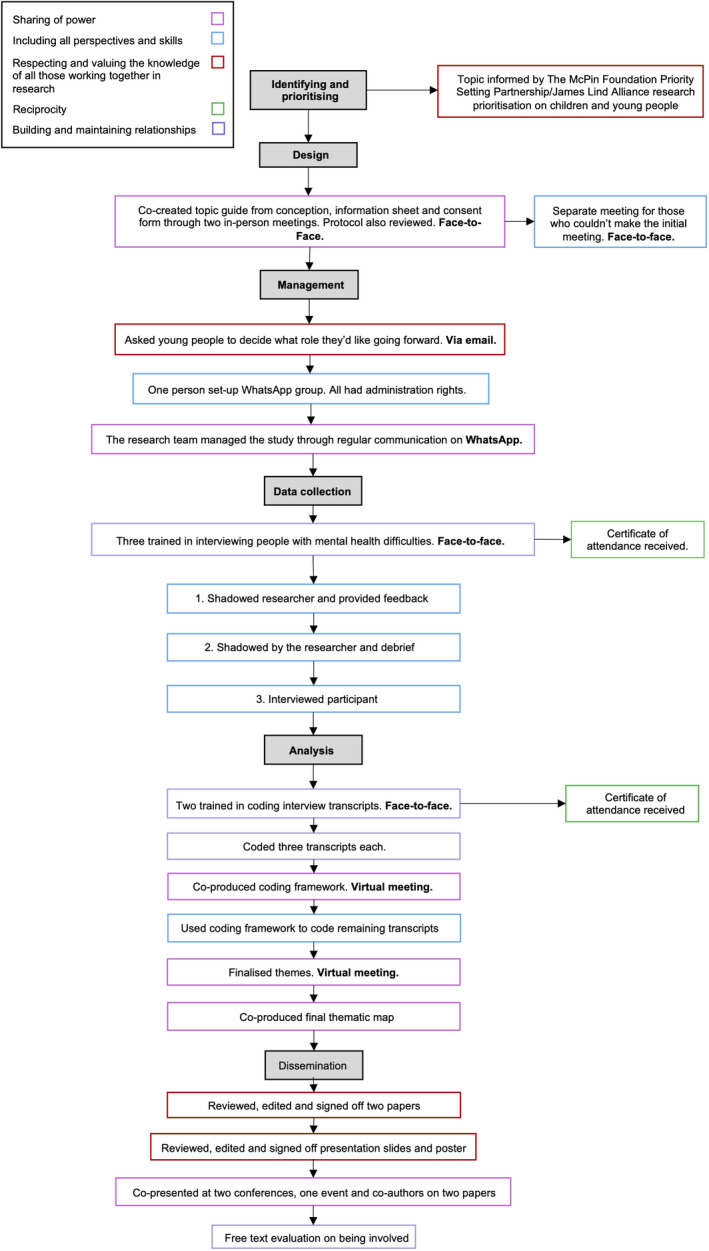
Flow chart showing young person involvement by principles of co‐production throughout all research stages

Initially, the YPAG reviewed the research question and protocol and co‐created the interview topic guide, information sheet and consent form (Figure 1) through two face‐to‐face meetings and further email exchanges.

### 2.3 Roles of involvement

The seven YPAG members were asked to choose which area of the research they would next like to be involved in (a) data collection and/or data analysis, (b) management, and (c) dissemination. All YPAG members were assigned the roles they requested—two were assigned to management, three to data collection and analysis and two to dissemination. However, three YPAG members dropped out from management and data collection and analysis, before roles commenced due to deteriorating health and potential breach of the expense policy. This breach is wider than the project and for legal reasons cannot be discussed in this paper.

All roles were re‐advertised but just one new person applied (resulting in five YPAG members) and was appointed two months after the project started. This person was involved in management, data collection, analysis and dissemination. The three young people assigned to data collection and analysis became embedded in the research project and were subsequently involved in management and dissemination and as such, described as co‐researchers (Figure 1).

### 2.4 Training

Three co‐researchers were trained in conducting interviews with people with mental health difficulties and coding interview transcripts. The training involved two, one‐day (9:30 am‐5 pm), face‐to‐face sessions. Co‐researchers were paid for their time (£150 each per session). Disclosure and Barring Check (DBS) were sought and approved for all co‐researchers. A full breakdown of the training process is provided in Appendix 2.

### 2.5 Data collection

The team co‐produced a working model that described the best approach for co‐researchers to interview participants in this study. As a team we decided on a three‐stage approach: (a) co‐researcher to shadow the lead researcher (LD) (with the participant's consent); (b) co‐researcher to then conduct the interview with LD present, and (c) co‐researcher to conduct the interview alone. Each co‐researcher was debriefed separately about what went well and what could be improved from both the perspective of the lead researcher and the co‐researchers, and there was time for questions. The co‐researcher and researcher were present in six interviews. Co‐researchers shadowed two, conducted three with the researcher present and conducted one alone, with the researcher in the next room for support if needed. A psychiatrist was available on site for safeguarding support throughout.

### 2.6 Analysis

Unfortunately, one of the co‐researchers informed the lead researcher that they could no longer be involved in the remainder of the project because of personal reasons. Two co‐researchers (CC and JJ) continued and analysed three transcripts each which were also coded by two researchers (LD and ML); the lead researcher (LD) was contactable for support. The co‐researchers coded their associated interview transcripts when possible. We co‐produced a thematic coding framework during a 2‐hour virtual meeting (Skype) which we then used to analyse the rest of the transcripts. We then co‐produced a thematic map over a further two virtual meetings: (a) the researchers wrote the themes and sub‐themes on a white board and we discussed these as a group; (b) all suggestions from co‐researchers were added to the board; (c) we again discussed these as a group; (d) we added, refined or removed themes/sub‐themes/links; and finally; (e) reached consensus on final themes and sub‐themes.

### 2.7 Dissemination

Four presentations (two posters and two oral) were co‐presented at national and international conferences.^25^, ^26^, ^27^, ^28^ The lead researcher (LD) produced presentation drafts and co‐researchers made comments and changes. This approach was utilized because of the limited amount of money dedicated to this level of dissemination involvement (four outputs) at grant projection and study planning stage. The co‐researchers were also in employment or education, which made it more difficult for co‐researchers to complete the first drafts which required substantial time commitment. We had a further 2‐hour meeting to decide together who would present each section and to practice the oral presentation. Both co‐researchers (CC and JJ) and the young person originally assigned to dissemination (KP), also contributed to two papers (the current paper and the main qualitative paper) as co‐authors. The two co‐researchers (CK and JJ) also reflected on, and evaluated, each stage of involvement and the support given during the project using questionnaires, free‐text responses and face‐to‐face discussion.

## 3 RESULTS

### 3.1 Principles of co‐production

Our study is described against the principles of co‐production (Box 1) across the research stages, from design to dissemination.

BOX 1 **Principles of co‐production^10^**

1. Sharing of power
2. Including all perspectives and skills
3. Respecting and valuing knowledge of all those working together on the research
4. Reciprocity
5. Building and maintaining relationships

#### 3.1.1 Sharing of power

Trust and rapport were developed between the members of the team by taking time early in the research process to get to know each other better. The researchers and PPI lead began by sharing their own experiences of mental health difficulties to help create a safe space. The co‐researchers then shared their own experiences. Moreover, our shared values and complementary working approaches (eg hard working, passionate for the subject, active listeners, empathetic) helped develop this rapport.

Sharing power and decision‐making between the young co‐researchers and lead researcher became more substantial after each research stage, as our trust and confidence in each other increased; co‐researchers’ ideas were listened to and acted on. For example, the young people took the lead in developing the topic guide questions. On suggestion of the co‐researchers, we carried out a lunchtime meet and greet at the clinical site for the research team to meet potential study participants, to encourage them to take part. One of the co‐researchers suggested we contact each other by WhatsApp and led on creating the group. This enabled everyone to be more easily involved in decision‐making, as contact could be made quickly and the information readily available and all stored in one searchable thread.

I felt very involved with the decision‐ making progress throughout the project. The decision‐making process was democratic, and we have often had the opportunity to review things before they are finalised and suggest changes. I think that the team, handled discussions very well and we were able to work well to achieve a good result. (CC)

Being a part of this team has been wonderful and extraordinary, and “empowered” is not a strong enough word for how I feel. Thank you for letting me be involved – this project has completely transformed how I view the world of research and technology, and I will be forever grateful :‐). (JJ)

I found the experience very rewarding, working closely with the co‐researchers to analyse the qualitative data provided me with alternative analytical perspectives. I believe this led to a more comprehensive and contextualised interpretation of the data, which would not have been possible without that collaboration. (ML)

#### 3.1.2 Including all perspectives and skills

Our YPAG involvement strategy aimed to include people of varied experiences in both mental health and technology. We were successful in appointing a diverse group of young people aged between 18 and 25 years old (similar to our research target age group) who had experience of most mental health conditions between them and some experience of conducting research. Whilst all were based in London due to budgetary restraints, we were mindful of diversity, including location in London, ethnicity, current occupation and physical disabilities. However, following discussion with the psychiatrist, we did not appoint people with severe current mental health difficulties because of ethical and safeguarding considerations. Having a psychiatrist involved in the project brought in another key perspective from the clinical frontline (SP).

All training and research documentation were presented in Plain English and in‐person so there were opportunities for questions. However, the young people felt there was not enough time given to explaining the different types of technologies when introducing the project and producing the topic guide, especially the less common ones (eg wearables).

We could have spent more time as a team going through previous examples of technologies. Not everyone had the same amount of information regarding these technologies and their relevance, and this makes for variable interview content. (CC)

#### 3.1.3 Respecting and valuing knowledge

In our case, respecting and valuing each other and therefore the knowledge of all of us working on the project came naturally, due to our similar values and characteristics. We were aware that we all came from differing, but equally valuable perspectives and created a safe space: agreeing on meeting principles; openly sharing personal experiences; and spending time to get to know each other before/after each meeting. There were strong support and communication as we talked via virtual meetings (Skype/FaceTime), a WhatsApp group, emails, and in person—in places and at times that suited everyone. We tried to accommodate every person's needs and preferences (eg around university and working hours). For example, some people could not attend one face‐to‐face meeting; therefore, a separate meeting was arranged for them. We also recognized team members brought different expertise. The co‐researchers requested research documentation to be first drafted by the researcher and then reviewed by the co‐researchers, as the researcher had previous experience but it would ensure the co‐researchers’ views were still integrated. Co‐researchers reported they felt appropriately supported and valued members of the team (Appendix 3).

Such a wonderfully collaborative working environment – I think we all worked really well together and valued and respected each other's opinions. (JJ)

#### 3.1.4 Reciprocity

Both the researchers and co‐researchers benefited from co‐production, recognized by the contribution that all team members made to the project. For the co‐researchers, this included obtaining new knowledge (about research, co‐production and the subject area); increasing confidence, gaining new skills (in interviewing, basic thematic analysis and evaluation); building on skills (group work, presentation and reflection); networking; and being financially recognized for time on the project. Those trained in data collection and analysis received a personalized certificate of attendance. The co‐researchers gained further recognition and exposure at conference presentations. They also co‐facilitated two workshops on co‐production, one filmed by West London NHS Trust and one interactive workshop at a national conference. The lead researcher (LD) produced a training package focused on conducting interviews and coding transcripts (in Plain English) which other researchers recognized and replicated. LD, ALJ and ML built on their knowledge of PPI, enriched their networks and gained invaluable experience of co‐producing a research project, including overcoming challenges such as time, budget and YPAG members leaving the project (Appendix 3).

Working with young people with past mental health difficulties throughout the whole research project – from design to dissemination – helped produce better informed research findings for our study. This experience was a big learning curve for me and definitely worth it. Going forward, I will use what I have learned to design PPI plans and research projects differently. It has made me always want to take time to do meaningful PPI! (LD)

#### 3.1.5 Building and maintaining relationships

We worked hard to build and maintain relationships by allowing time to build trust. Initially, we met everyone face‐to‐face and started each meeting with an informal period where we talked about our breaks, weekends and latest news. This helped the group to bond on a personal level, feel comfortable to speak up and break down any predefined power differentials. This was particularly apparent with the co‐researchers because responding quickly to each other through WhatsApp helped to show that we were all prioritizing the project and each other. These supportive relationships have continued past the lifespan of the project. Between March 2018 and July 2019, there were 494 WhatsApp messages and 23 images sent between us. In contrast, this was not the case for the other young people who were not involved in the data collection and analysis stages. This was because role activity was intermittent and although they were given ad hoc project updates, there was not as much time to build as strong relationships.

A new co‐researcher was recruited two months after the project started due to another potential co‐researcher's health deterioration. This could have delayed the project because we thought it would take time to build trust and relationships, integrate them into the team and explain the project and progress so far. However, this was not the case. We took time appointing someone who would match the role to collect/analyse data. The new person was introduced to everyone at the first face‐to‐face training session, and with the YPAG, we created a safe space, by allowing time to get to know each other. We set principles for the meeting (eg about confidentiality, being non‐judgemental and active listening) and everyone was invited to speak about their mental health experience (if they felt comfortable). This allowed the new young person to open up about their own experiences and blend in well with the group (Appendix 3).

### 3.2 Reporting reflections that capture the impact on the research, the researchers and co‐researchers

PPI impact was demonstrated in the design, data collection, analysis and dissemination stages. The co‐researchers co‐created the topic guide. The resulting guide was written in a way that young people could understand, was appropriate for the audience and community validity was evident.^18^ In the data collection, co‐researchers developed rapport with the interviewee quickly (eg interviewee 1, ‘you know what I mean!’). They made the interview feel like a conversation, humanizing the person and the situation, and understanding specific terminology. Consequently, they used appropriate probing on certain areas because they knew what the interviewee meant (eg ‘your Bipolar, go back [to talk] about the mania’). In contrast, the lead researcher would have missed this. However, personal experiences sometimes impacted on the level of probing. For example, sometimes a co‐researcher used leading questions because of their mutual mental health experience; they were invested in the conversation. This mainly happened in the first interview, when the researcher shadowed them and this improved each interview, after the self‐reflection and feedback sessions.

**FIGURE 2:**
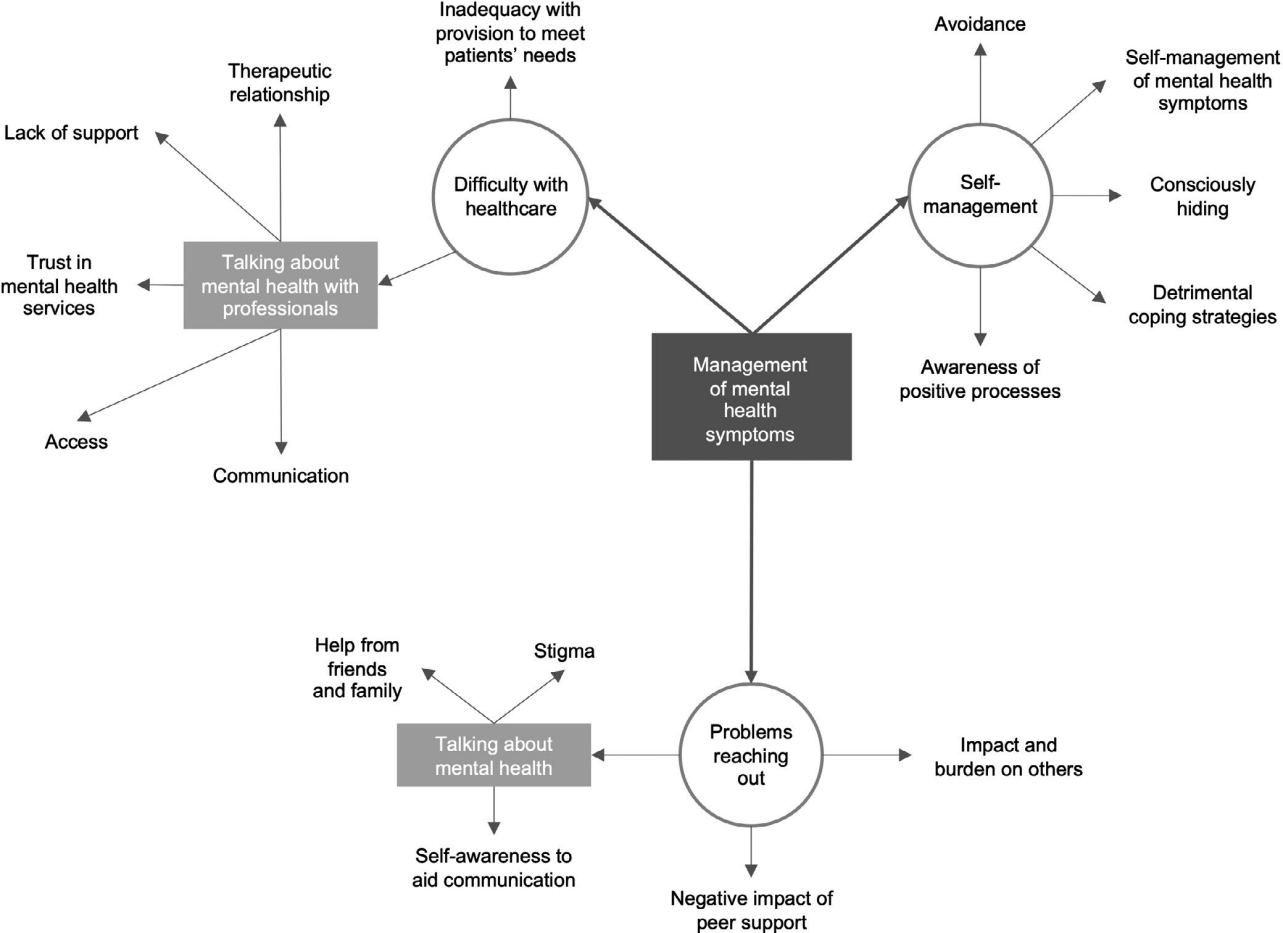
Section of thematic map before co‐researcher involvement

**FIGURE 3:**
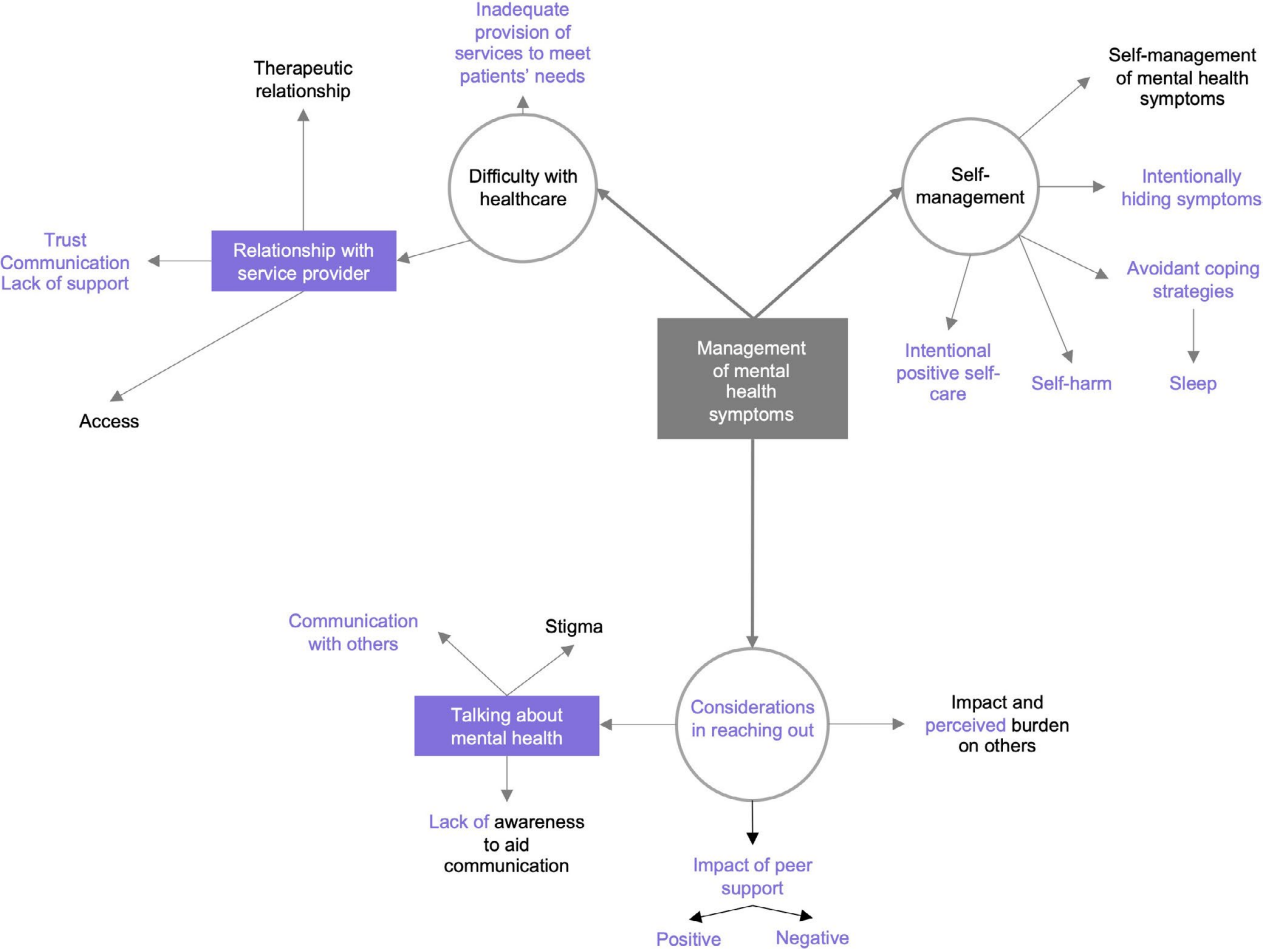
Section of thematic map after co‐researcher involvement

The impact of working with the co‐researchers to co‐produce a transcript framework and thematic map was evident in the data analysis. Figures 2 and 3 display the thematic map prior to (Figure 2), and after (Figure 3) co‐researcher input (Purple font reflects co‐researcher comments/changes). For example, co‐researchers changed the specific ‘negative impact of peer support’ sub‐theme to include both negative and positive impacts. Significant changes were made to wording of themes and sub‐themes and theme linkage. Sub‐themes were merged or separated based on the co‐researchers’ perspectives. For example, *issues in reaching out* were changed to *considerations of reaching out*, to reflect both positive and negative consequences.

Involvement of co‐researchers has delivered impact through dissemination at two important levels. Firstly, they contributed to dissemination of this research at international and national conferences and in international journal publications, and secondly, they shared their experience of the PPI process. This was shared from the front line, to clinical and academic audiences at conferences to public audiences that were young or new to PPI, and who may have wanted to follow in their footsteps.

## 4 DISCUSSION

### 4.1 Main findings

To our knowledge, this is the first paper to describe the process of co‐producing a qualitative mental health research study with young people, in line with the principles of co‐production. The relationships created and maintained throughout the project were vital to the study's success. Indeed, involving the same co‐researchers throughout the whole project, and not intermittently, translated into meaningful involvement and true co‐production. Their ability to develop rapport quickly with interviewees, probing deeper in interviews and understanding of terminology, was evidential impact on the research. However, our different experiences and backgrounds meant that at times we had conflicting priorities. For example, the lead researcher's priorities were as follows: rigorously collecting data that was needed to answer the research question; sticking to the topic guide (not going off‐track); and not asking leading questions to the participants. Although the co‐researchers were trained and aimed to do this, they had a greater tendency to reassure the participant and share their personal experiences, which could cause them to lead the participants and potentially affect the integrity of the data. This was managed by debriefing the co‐researchers on the need to balance building rapport with efficient data collection.

Furthermore, true co‐production takes time. Every effort was made to co‐produce the project in its entirety and to address power imbalances between the researcher and co‐researchers. Sharing power was made more difficult by the fact the lead researcher had to continue to maintain overall responsibility of the project including organizing PPI activity, resources, planning logistics for the interviews, meetings and events, as well as remaining responsible for the co‐researchers’ safety, finances and potential errors. This was unavoidable in an academic environment and with the budget available. Moreover, building trust and relationships, collective decision‐making and support, demanded dedication from both the researcher and co‐researchers and took up more time/budget than was originally predicted. Despite best efforts to plan, interactions with people and tasks will either (a) take up more time than expected; or (b) will require additional funds due to unexpected delays or events. Nevertheless, reciprocity was evident throughout, as co‐researchers gained new knowledge, skills and recognition from the University, PPI community and their peers. The researchers gained valuable experience of co‐producing a research project, overcoming challenges and also gained recognition from the academic and clinical communities.

INVOLVE has reported on six studies against the principles of co‐production.^12^, ^13^ To our knowledge, no other qualitative study involving young people with mental health difficulties has evaluated their study against the five principles of co‐production.^10^ However, a recent narrative review found 17 health research studies that involved patients as co‐researchers throughout the research process.^9^ All of these used qualitative methodology but only six took place in the UK and only three related to mental health. Moreover, only one of these studies had patient involvement or co‐production as its primary focus. Worryingly, the other studies that did centre on this (but from other topic areas, for example cancer and learning disabilities) had issues with transferability, relevance of findings and strength of impact. There is a limited number of studies that involved young co‐researchers (18‐25 years). The review authors argue that studies that do involve them, do not help advance knowledge.

Whilst we agree that working with co‐researchers is challenging because of our reflections above, our study used a rigorous assessment against the principles of co‐production that captured the impact on a research project, but also on the researcher and co‐researchers themselves. Co‐producing our study with young people offered a deeper understanding of the findings and terminology that would not have been achieved without them.

### 4.2 Future co‐production recommendations

Building on other recommendations,^6^, ^7^ we have several recommendations that can be applied to co‐production with young people who have experience of mental health difficulties, and more widely. We would recommend offering appropriate clinical support for young people with experience of mental health difficulties and involving several co‐researchers, in case of drop out (eg due to situation or health changes). Whilst there was group consensus of the need for the researcher to act in a ‘leader’ role (ie to have overall coordination and oversight of the project), we suggest potential honorary researcher status could be given to the co‐researchers, which could mean they could take on, or share, this role with the researcher, depending on budget and co‐researcher's desire/capacity/time. We also suggest considering young people to co‐design and co‐deliver the training to co‐researchers to further reduce power restraints.

Moreover, to build rapport within the team and reduce power imbalances, we recommend: (a) matching the person to the role, ensuring members know what to expect and what their roles are, (b) meeting in a place and time that is appropriate for young people, (c) creating an informal, light‐hearted environment from the start, (d) dedicating time to get to know each other beyond work, (e) bringing refreshments (eg tea/coffee, cake/sweets and healthy snacks) to aid concentration levels and make it a social experience, (f) creating a safe space for open discussion, (g) allowing for breaks, fresh air and ensure the activities are interactive, (h) ensuring strong communication and providing support, and (i) asking for and listening to feedback.

A flexible budget is needed for meaningful co‐production. We suggest using the INVOLVE calculator to predict PPI costings but also have extra contingency fund for tasks not originally costed.^21^ In the future, flexibility should also be acknowledged by funders, reviewers and governing bodies as currently this is not the case. For example, some suggestions by young people with lived experience were only put forward after the project had been funded and started (eg the meet and greet event). In addition to the training schedule (Appendix 2), the co‐researchers in particular recommend more practice in conducting interviews. For example, it would be helpful to provide the opportunity for the co‐researchers to practice the interviews on a different day with feedback (as we did not monitor if they practiced with friends) or provide a researcher to give feedback (Box 2). A refresher session could also be offered (with questions and answers), to increase their confidence and skills. Furthermore, we recommend capturing all reflections, dates, notes and feedback within an electronic notebook (eg Evernote).

BOX 2 **Key learning to take forward**

1. Match the person to the role (eg through skills, experiential knowledge^22^ and passion) and set expectations.
2. Have several young people included throughout the study and allow for flexible timelines and budget.
3. Take time getting to know each other and create a safe space for open discussion, which helps to build relationships.
4. Remain in regular contact (in ways suggested by young people), which helps to build trust, address power imbalances and allows for collective decision‐making.
5. Recognize that some roles may only be done by the researcher/clinician and give strong training/support (including clinical support) to co‐researchers doing new research activities, to ensure data is rigorous.

### 4.3 Limitations

We evaluated our work against the five principles of co‐production and provided reflections from the co‐researchers (CC and JJ) and researchers (LD and ML). Involving only one male co‐researcher may have negatively impacted upon perspectives across both genders. The psychiatrist's reflections were also not reported. This was mainly because building relationships and sharing power was primarily between the co‐researchers and lead researchers. In the future, we will attempt to record interactions between all team members throughout the research process. The article also provides recommendations that are primarily addressed to those undertaking mental health and qualitative research; hence, transferability might be limited. Nevertheless, most recommendations are centred on achieving the five principles which are universal. Additional work is needed to evaluate co‐production in quantitative and mixed methods studies, across different populations and areas other than mental health.

## CONFLICT OF INTEREST

All authors have completed the Unified Competing Form and declare: no support from any organization for the submitted work; no financial relationships with any organizations that might have an interest in the submitted work in the previous three years; and no other relationships or activities that could appear to have influenced the submitted work.

## AUTHOR CONTRIBUTIONS

LD, ALJ, CK and JJ made substantial contributions to conception, design and paper protocol. LD, ALJ, CK, JJ, KP, ML, SP and PA made substantial contributions to the acquisition and interpretation of data. All authors have been involved in drafting manuscript or revising it critically for important intellectual content and approved the final manuscript.

## ETHICAL APPROVAL

Ethical approval was not needed for this reflection point within the qualitative mental health study. It should not be read as a research paper.

## Data Availability

Data sharing is not applicable to this article as no new data were created or analysed in this study.

## Appendix 1

### GRIPP2 REPORTING CHECKLIST

**Table:**

Section and topic	Item	Reported on page No
*Section 1: Abstract of paper*		
1a: Aim	Report the aim of the study	1
1b: Methods	Describe the methods used by which patients and the public were involved	1
1c: Results	Report the impacts and outcomes of PPI in the study	1
1d: Conclusions	Summarize the main conclusions of the study	1
1e: Keywords	Include PPI, ‘patient and public involvement’, or alternative terms as keywords	2
*Section 2: Background to paper*		
2a: Definition	Report the definition of PPI used in the study and how it links to comparable studies	3
2b: Theoretical underpinnings	Report the theoretical rationale and any theoretical influences relating to PPI in the study	3
2c: Concepts and theory development	Report any conceptual models or influences used in the study	3
*Section 3: Aims of paper*		
3: Aim	Report the aim of the study	4
*Section 4: Methods of paper*		
4a: Design	Provide a clear description of methods by which patients and the public were involved	4‐6
4b: People involved	Provide a description of patients, carers and the public involved with the PPI activity in the study	4
4c: Stages of involvement	Report on how PPI is used at different stages of the study	4‐6
4d: Level or nature of involvement	Report the level or nature of PPI used at various stages of the study	4‐6
*Section 5: Capture or measurement of PPI impact*		
5a: Qualitative evidence of impact	If applicable, report the methods used to qualitatively explore the impact of PPI in the study	7‐12
5b: Quantitative evidence of impact	If applicable, report the methods used to quantitatively measure or assess the impact of PPI	N/A
5c: Robustness of measure	If applicable, report the rigour of the method used to capture or measure the impact of PPI	N/A
*Section 6: Economic assessment*		
6: Economic assessment	If applicable, report the method used for an economic assessment of PPI	N/A
*Section 7: Study results*		
7a: Outcomes of PPI	Report the results of PPI in the study, including both positive and negative outcomes	7‐12
7b: Impacts of PPI	Report the positive and negative impacts that PPI has had on the research, the individuals involved (including patients and researchers), and wider impacts	11‐12
7c: Context of PPI	Report the influence of any contextual factors that enabled or hindered the process or impact of PPI	7‐12
7d: Process of PPI	Report the influence of any process factors that enabled or hindered the impact of PPI	7‐12
7ei: Theory development	Report any conceptual or theoretical development in PPI that have emerged	N/A
7eii: Theory development	Report evaluation of theoretical models, if any	N/A
7f: Measurement	If applicable, report all aspects of instrument development and testing (eg validity, reliability, feasibility, acceptability, responsiveness, interpretability, appropriateness and precision)	N/A
7g: Economic assessment	Report any information on the costs or benefit of PPI	14
*Section 8: Discussion and conclusions*		12‐15

## Appendix 2

### STEPS TAKEN TO TRAIN AND SUPPORT YOUNG PEOPLE WITH PAST MENTAL HEALTH DIFFICULTIES IN CONDUCTING INTERVIEWS AND CODING TRANSCRIPTS

**Table:**

Step	Detail
Training preparation (for session 1 and 2)	Pre‐read documentation on the project prepared and sent to co‐researchers Doodle poll set‐up, sent out and training dates confirmed Rooms booked and catering ordered for all day (coffee/tea/water—AM/PM, lunch, cakes and healthy snacks) Presentation and key points about interviewing prepared by lead researcher (LD) and approved by PPI lead (ALJ)
Training session 1 (Conducting interviews)	Initial introductions, learning objectives and expectations for the day were discussed Discussed everyone's background and skills and a brief recap on the research project was conducted Main considerations for interviewing people with current mental health conditions in an interactive session were discussed Researchers performed role plays of conducting an interview (eg including potentially challenging situations) to help the co‐researchers to gain tools and confidence Co‐researchers took part in role play and took turns as interviewee, interviewer or shadowing the interview (gave specific feedback, alongside feedback from the researchers) Questions and answers session conducted
Feedback, actions and homework	Researchers/co‐researchers gave feedback via email and held questions and answers sessions Co‐researchers were given take away materials including notes, extra tips and reflection from the training day (sheet to refer to) Co‐researchers were asked to spend time revising the topic guide and practising interviewing with a friend Each co‐researcher was given certificate of attendance Researcher was available to be contacted with any questions
During the interviews	Co‐researcher shadowed researcher in real interview; then, researcher shadowed co‐researcher Researcher and clinician were onsite for any support for co‐researchers before/during/after interviews, including a debrief after each interview with self‐reflection and feedback from researcher Researcher was contactable at all times throughout interview days and in‐between interviews
Training session 2 (Coding transcripts)	Reflection on being a co‐researcher and interviewing people was discussed An initial impression of the data collected in the interviews was discussed Went through working example of a thematic analysis using previous qualitative study Introduction to basic thematic analysis and practice session on first two steps (1) familiarization and (2) initial coding a transcript (matched to an interview they have conducted when possible) was conducted On‐site support from lead researcher, PPI lead and PhD student was provided Questions and answers session and feedback
Feedback, actions and homework	Researchers/co‐researchers give feedback via email and hold a Q and A session Take away materials given to co‐researchers including quick guide to thematic analysis Each co‐researcher given certificate of attendance Co‐researchers asked to code another two transcripts each Researcher was available to answer any questions

## Appendix 3

### ADDITIONAL QUOTES SUPPORTING THE PRINCIPLES OF CO‐PRODUCTION

#### Quote 1

‘The support was really good overall. I felt like a valued member of the team and I was well looked after’ (CC)

#### Quote 2

‘I enjoyed the initial session of proofreading the topic guide and information sheet as a group. It felt like my edits were valuable and it was really rewarding to see that something I said or contributed ended up being in the final product’ (JJ)

#### Quote 3

‘…I think it's an enjoyable and interesting experience to get involved in a project like this from any background. I think that in a sense it gives more context to one's experiences, and it does to some extent make the things that one might have experienced feel like they at least have a use in the world. In addition to this I feel I’ve gained useful people and teamwork skills and also training in research methods that will be useful to me in my degree and hopefully elsewhere in my career’ (CC)

#### Quote 4

‘I have nothing but good things to say about the process and the team that we have worked in the process has been amazing and informative and overall a great experience for me. Although the process definitely, brought up some memories for me, I would not describe this as being negative, in some ways it just provided a new perspective on my own experiences and those of others. In addition, I felt that it was useful to be able to apply what I have learnt from my own experiences, and Lindsay was good at being aware of the potential impact that doing the research might have on us and giving me a positive perspective on reflections’. (CC)
